# Home Treatment

**Published:** 1911-11-04

**Authors:** 


### HOME TREATMENT.

THE first question which has to be answered when a case of insanity appears is this: Can the case be treated at home, or must it be sent away? Institutions for treating the acutely insane are a necessity at the present day as a matter of economy, because the expense of looking after a case is greater when it is in single care than when it is treated with a number of others. The answer to the above question, then, depends primarily on the financial position of the friends of the patient. It is true that in some cases an institution will be necessary for care and treatment, but unless the means of the patient permit, the experiment of home treatment cannot be made at all before certifying.

The essentials of home treatment in acute cases will be: (1) A room for the patient, away from any traffic through the house, which can be made secure enough to safeguard the patient during any excited, impulsive, or suicidal mood; (2) some space for proper exercise in the open air, where the patient will not annoy, nor be annoyed by, other people; (3) sufficient nurses to look after him by day and by night. The advantages of home treatment will be the probable escape from certification and its stigma, the direct observation of friends and relatives of the patient, and, in quiet cases, the avoidance of any locks and keys, which are bound to be irksome to the patient from the feeling of restraint that is engendered by being locked into a room.

There is an important fact which must never be lost sight of in home treatment, and that is the absolute impossibility of foretelling, even roughly, the probable duration of the attack. Medical men who have had but little actual experience in treating the insane do not seem to realise this impossibility, and much unfairness is often done to medical officers of institutions for the insane by the practitioner saying to the friends that the patient will be, or ought to be, well in such and such a time; and in self-defence the medical officer has to point out plainly how unjustifiable is such a statement. What turn events may take, even in the mildest case, it is impossible to prophesy, and the chances of a case hanging fire or completely changing its type, *e.g.* from mania to melancholia or *vice versa*, in the time stated are just as great as for recovery. Since there can be no grounds upon which to base any statement as to length of time necessary for recovery except actual experience, such statements should be very cautiously made. All that it is possible to say about any case is whether, in the doctor's opinion, the case is likely to get better or not, and what is the average length of an attack in such cases (if he knows it), mere guessing or romancing on the subject being not only useless, but possibly harmful. The bearing that this uncertainty of length of attack has upon the financial aspect of home treatment scarcely needs explanation.

